# Recurrent pancreatitis by invasive IPMN in young patient: case report and review of management strategies

**DOI:** 10.1097/RC9.0000000000000229

**Published:** 2026-09-24

**Authors:** Alberto Torrisi, Roberto Cantella, Danilo Paolo Cannizzaro, Guido Basile, Martina Reina, Giuseppe Angelo Reina

**Affiliations:** a Department of General Surgery and Medical-Surgical Specialism, Post-graduate School in General Surgery, University of Catania, Italy; b General and Emergency Surgery Department of Paternò-Biancavilla, ASP Catania, Catania, Italy

**Keywords:** case report, IPMN, laparoscopic pancreatectomy, recurrent pancreatitis, worrisome features, young age

## Abstract

**Introduction:**

Intraductal papillary mucinous neoplasm (IPMN) is a cystic lesion of the pancreas connected to the ductal system. It carries a risk of malignant transformation, especially in the main-duct form (MD-IPMN). Most cases occur in patients older than 60 years, but early presentation is possible and may indicate a genetic condition that should be investigated. The evolution of diagnostic techniques and updates to guidelines allow risk stratification and management based on high-risk stigmata and worrisome features.

**Case presentation:**

A 31-year-old woman presented with acute pancreatitis. Clinical and radiological evaluations led to the diagnosis of main-duct IPMN. After surgical resection, high-grade dysplasia with diffuse foci of micro-invasive carcinoma was found and graded as PanIN-3. A genetic evaluation was recommended in light of the combined findings of young age and aggressive histopathology.

**Discussion:**

This rare clinical case highlights the possibility of early-onset malignant conditions. A thorough diagnostic workup in young patients with pancreatitis, especially if of non-lithiasic origin, remains essential for correct therapeutic decision-making. It has been demonstrated that survival rates vary significantly between low-grade and invasive histopathologic types. This underscores the importance of early identification and timely treatment. Laparoscopic pancreatectomy has proven to be a valid approach for achieving a favorable prognosis.

**Conclusion:**

This clinical case emphasizes the role of early diagnosis and appropriate therapeutic management. In aggressive forms, it highlights the need for precise surgical intervention and long-term multidisciplinary surveillance. This case contributes to the evolving understanding of IPMN in younger populations and supports the ongoing refinement of guidelines.

## Introduction

Intraductal papillary mucinous neoplasm (IPMN) is a pancreatic lesion that originates from the pancreatic ducts and secretes mucin. IPMNs are a type of cystic pancreatic neoplasm that encompass a spectrum from benign adenomas to invasive carcinomas^[^1^]^. Based on ductal tree involvement, IPMNs are classified into Main-duct IPMN (MD-IPMN), Branch-duct IPMN (BD-IPMN), and mixed-type^[^2^]^. MD-IPMNs have main duct dilation greater than 10 mm and a higher risk of becoming high-grade dysplasia or invasive carcinoma, with malignancy rates reaching up to 50%^[^3^]^. Computed Tomography and Nuclear Magnetic Resonance Imaging have improved the detection of pancreatic cystic lesions, with prevalence rates ranging from 13% to 18%^[^4^]^. Pancreatic cystic lesions typically occur in individuals aged 60–70 years; however, among IPMNs, MD-IPMN carries the highest malignancy risk^[^5^]^. Age represents a risk factor for disease occurrence, with a reported prevalence of 2% in patients under 39 years of age and 26% in the elderly^[^4^]^. IPMNs are usually asymptomatic but can cause abdominal pain, jaundice, weight loss, diabetes, and pancreatitis^[^1^]^. Recurrent pancreatitis is recognized as the main, or more often the only, manifestation of IPMN. Approximately 12% to 67% of IPMN patients experience at least one episode of acute pancreatitis^[^3^]^, which is caused by pancreatic duct obstruction due to excess mucin production. Therefore, detailed ductal evaluation is essential^[^6,7^]^.

HIGHLIGHTS

- Recurrent pancreatitis can be the first sign of MD-IPMN, even in younger patients.
- Timely identification of IPMN is essential for appropriate therapy.
- Current guidelines emphasize the need for surgery for IPMNs with malignant potential.
- In a young patient with multifocal IPMN, genetic evaluation is recommended.
- Our case underscores that risk stratification is crucial, including in young patients.

Current guidelines categorize IPMNs based on “high-risk stigmata” and “worrisome features” to facilitate therapeutic decisions^[^2,8^]^. High-risk stigmata include jaundice associated with pancreatic cysts, an enhancing solid component or mural nodule of ≥5 mm, and dilation of the main duct ≥10 mm, which generally necessitate surgical resection^[^2^]^. Worrisome features, such as cyst size ≥3 cm, increased main duct size (5–9 mm), non-enhancing mural nodules <5 mm, abrupt duct caliber change, elevated serum CA 19-9, new-onset diabetes mellitus, and pancreatitis associated with IPMN, warrant further evaluation via endoscopic ultrasound and cytology^[^2^]^. The guidelines strongly suggest a more aggressive surgical approach for MD-IPMN, considering the high prevalence of advanced pathology^[^2,8^]^.

Younger patients, including those under 40, may occasionally develop the condition. In such cases, early recognition and precise risk stratification become crucial because even seemingly low-risk cysts can harbor malignancy. Additionally, when these are combined with multifocal disease or high-grade dysplasia, genetic evaluation should be recommended due to the potential for hereditary predispositions^[^9^]^.

The present case describes a 31-year-old woman with recurrent pancreatitis and a radiologic finding of an MD-IPMN. The present work has been reported in accordance with the SCARE criteria^[^10^]^.

## Case presentation

A 31-year-old woman presented to the emergency department in September 2024 with severe epigastric pain and nausea. She denied alcohol abuse, reported having recently given birth, and had an unremarkable medical history except for an incidentally diagnosed pancreatic cyst 2 years prior, which was not investigated further.

Laboratory examination revealed an elevated serum amylase level (1200 U/L) with normal liver function tests and triglyceride levels. Contrast-enhanced CT showed acute interstitial edematous pancreatitis with peripancreatic fluid extending into the retromesenteric and subhepatic regions and a 2.0 × 1.8 cm hypodense lesion in the pancreatic tail; no gallstones were identified (Fig. 1A).

**Figure 1.**
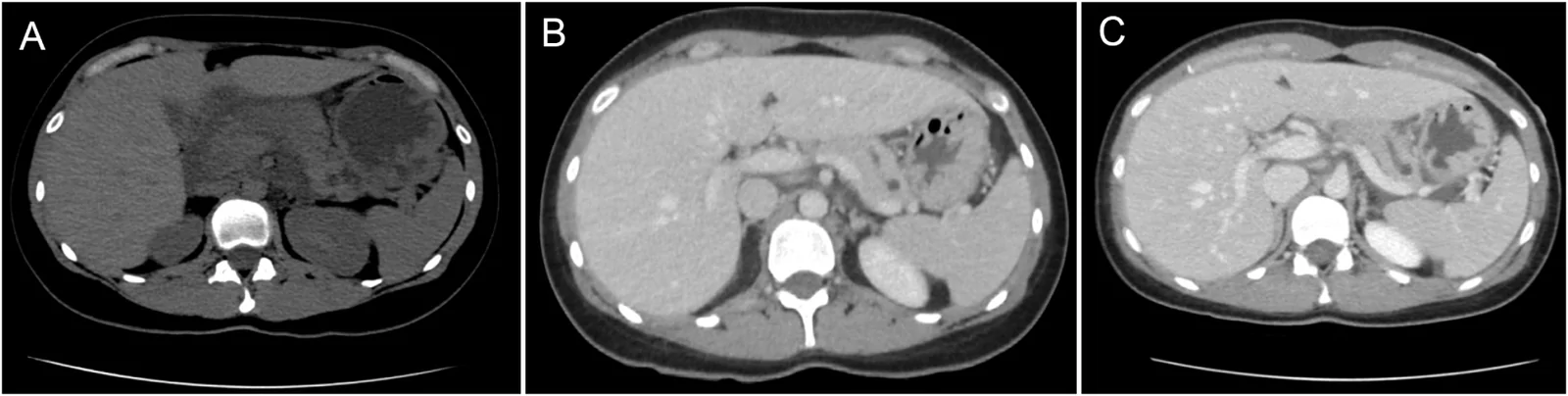
(A) CT of 9–24 (first documentation), (B) CT of 10–24 (after resolution of pancreatitis), (C) CT of 12–24 (recurrence of pancreatitis with enlargement of lesions).

After 5 days of intravenous fluids, analgesics, pancreatic secretion inhibitors, and antibiotics, she was discharged with planned follow-up. A subsequent CT scan showed resolved inflammation with 5 mm main pancreatic duct dilation and a 17 mm cystic lesion, suspicious for MD-IPMN. Confirmatory MRI in October 2024 identified a 15 × 11 mm bilobed cystic lesion communicating with an 8 mm dilated main duct, without enhancing nodules; serum CA 19-9 was normal. Endoscopic ultrasound was not performed, as imaging characteristics and clinical presentation were sufficient to guide management. Six-month imaging follow-up was scheduled.

In December 2024, the patient returned with recurrent abdominal pain. Emergency CT revealed acute pancreatic inflammation and enlargement of a 25 mm cyst (Fig. 1C). Progressive cyst growth and recurrent non-biliary pancreatitis (key worrisome features according to current guidelines) indicated surgical intervention^[^2,8^]^.

Laparoscopic distal pancreatectomy was performed in January 2025. The procedure involved mobilizing the distal pancreas, identifying and dividing the splenic vasculature, and transecting the pancreatic body with a stapler, achieving complete resection of the body and tail and spleen^[^11^]^ (Figs 2A–D and 3). Operative time was 180 minutes with 200 mL blood loss; one drain was placed, and the postoperative course was uncomplicated (Clavien–Dindo grade 0). Oral intake resumed on postoperative day 3.

**Figure 2.**
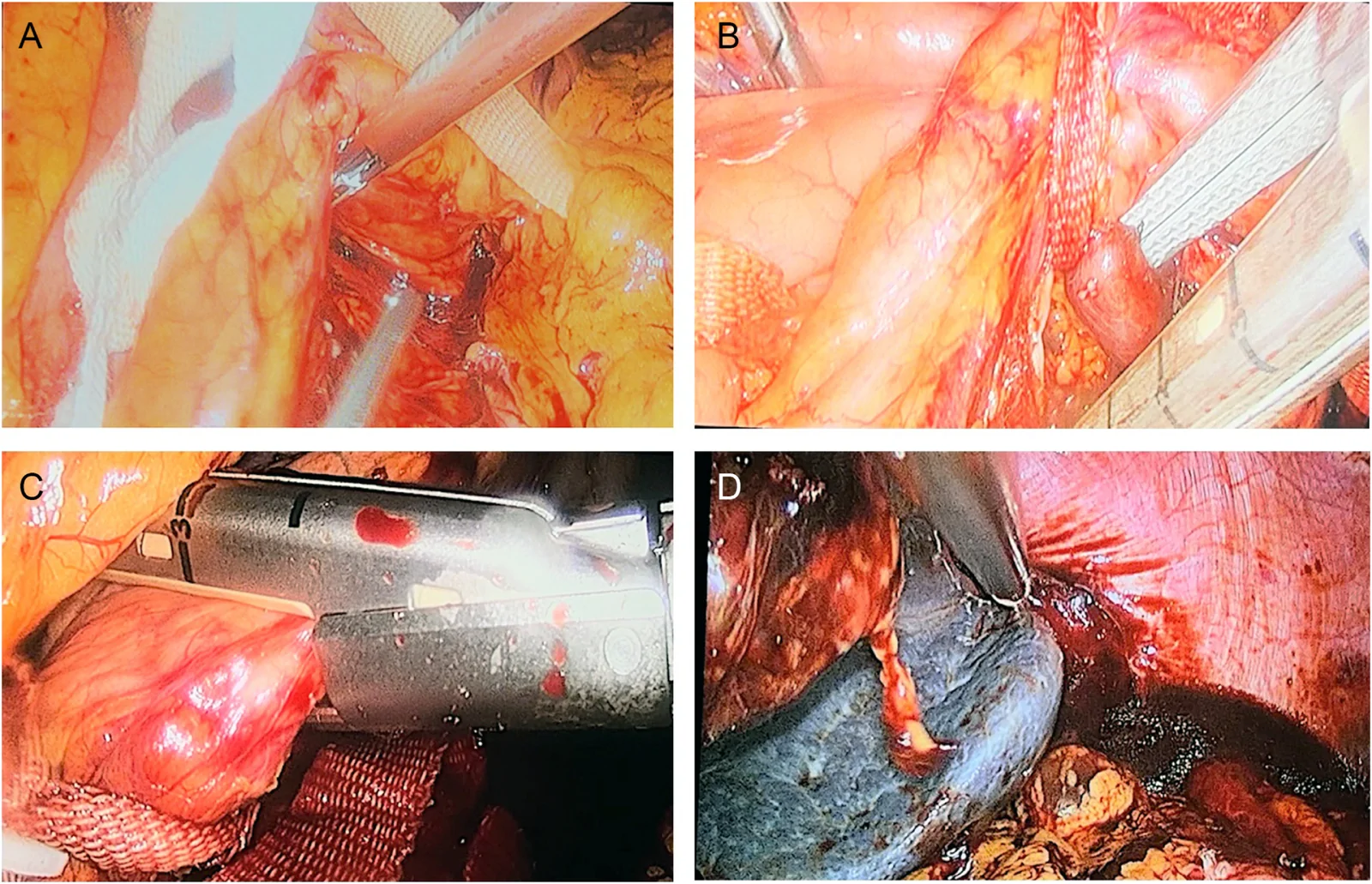
(A) Mobilization of the distal pancreas; (B) transection of the splenic vessel; (C) section of the pancreatic body and tail; and (D) mobilization of the splenic ligaments.

**Figura 3.**
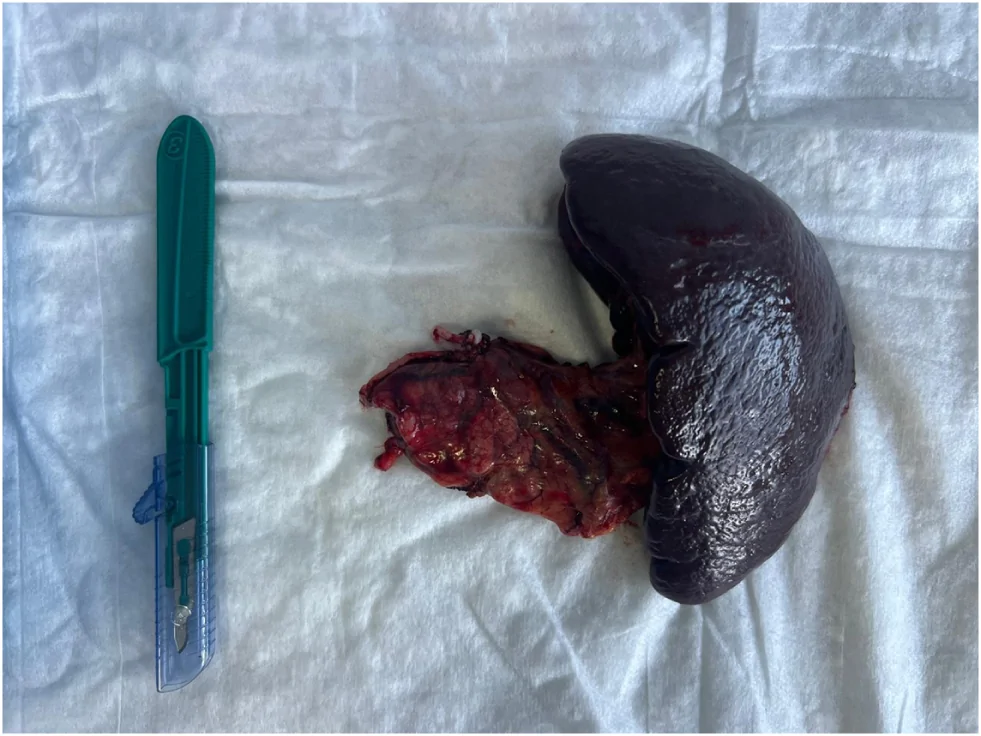
Surgical specimen.

Histopathology revealed a pancreatobiliary-type MD-IPMN with focal high-grade dysplasia and a 0.76 mm microinvasive adenocarcinoma, accompanied by multifocal PanIN-3 lesions in the surrounding parenchyma. Eight resected locoregional lymph nodes were negative for metastases. Resection margins were negative (R0). Final staging: TNM pT1bN0M0 (Fig. 4).

**Figura 4.**
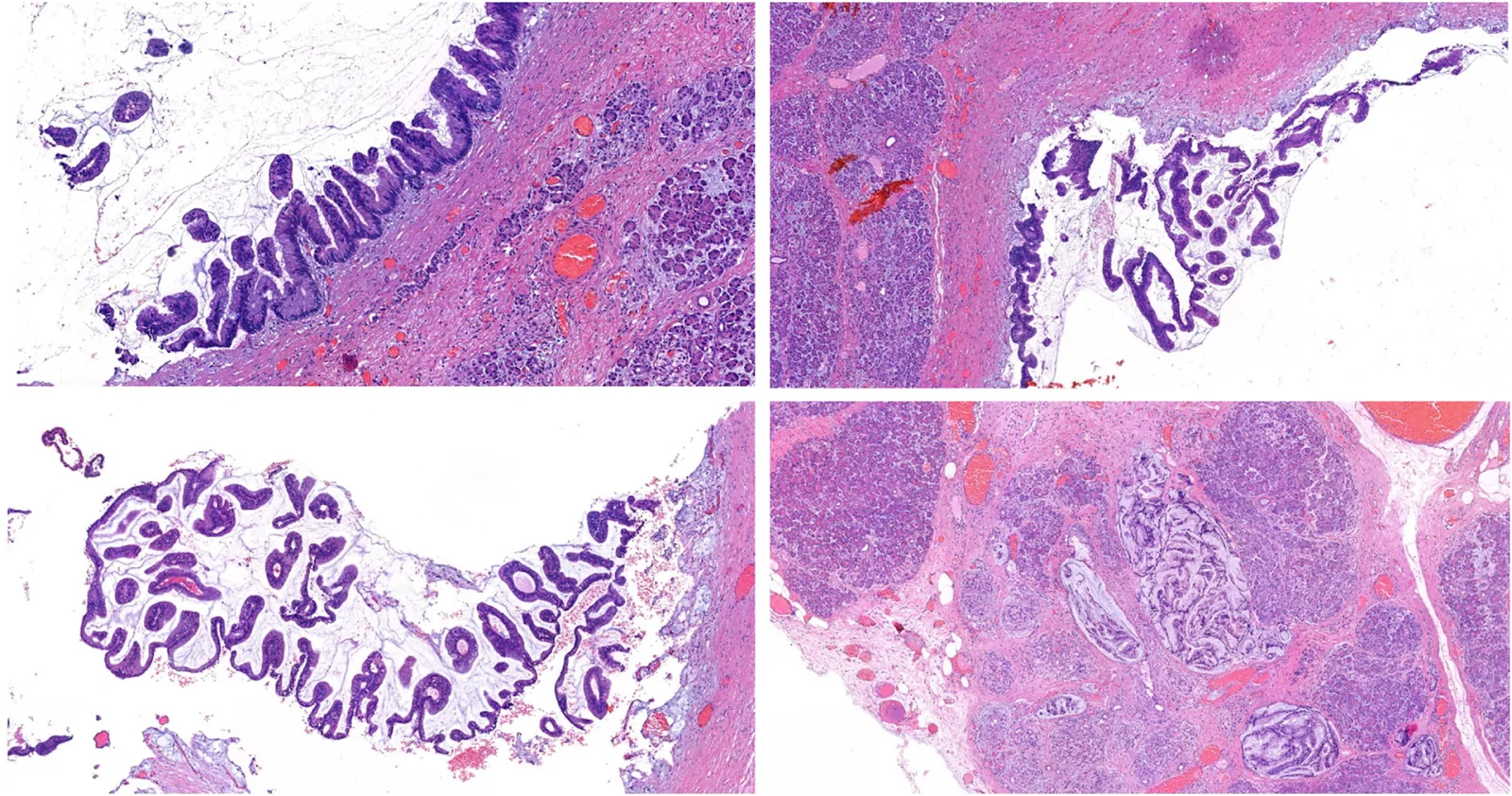
Histopathological features in hematoxylin and eosin staining at 10× magnification.

The combination of early-onset disease with invasive histology raised concern for a hereditary predisposition^[^9^]^. Genetic counseling and testing were initiated but did not detect any hereditary mutations, making the case even rarer.

The patient was discharged on postoperative day 10 and entered a structured surveillance protocol with imaging at 3 and 12 months, which demonstrated no recurrence to date, and continued multidisciplinary follow-up is planned annually.

## Discussion

IPMNs are associated with malignant potential, particularly in MD-IPMN, though they predominantly affect individuals in their sixth to seventh decades^[^2,9,12^]^. This case is remarkable for its atypical early presentation with advanced, aggressive pathology in a 31-year-old patient^[^2,9,12^]^.

Some surgical series have reported that only 5% of patients with resected IPMN are under 50 years old; these younger individuals often exhibit better survival outcomes, yet they remain at significant risk^[^13^]^.

According to these findings, more attention should be paid to the diagnosis of pancreatitis in young patients.

Early diagnosis and timely therapy are key points in pathology management^[^12,13^]^. A Finnish nationwide retrospective study demonstrated 10-year survival rates of 72.5% in low-grade cases compared to 23.1% in invasive cases^[^12^]^.

A recent French study showed that performing resection at an earlier stage is linked to a ten-year survival rate of 62.0% among IPMN patients with invasive malignant features, further supporting the efficacy of adequate intervention^[^13^]^. In our case, laparoscopic distal pancreatectomy had a good outcome with an R0 resection, and biopsy analysis revealed microscopic invasion (0.76 mm) and negative loco-regional lymph nodes.

Up to 67% of IPMN cases are linked to at least one episode of pancreatitis^[^2^]^. Pancreatic duct obstruction can lead to enzymatic hyperactivation and parenchymal inflammation; therefore, comprehensive evaluation using CT and/or MRI/MRCP is crucial for early detection^[^4^]^. In this patient, progressive cyst dilation from 17 mm to 25 mm correlated with symptomatic exacerbations, fitting the “worrisome features” category. Notably, this case achieved a surgical indication based on worrisome features (recurrent pancreatitis and cyst growth), despite the main duct diameter remaining <10 mm. Current guidelines recommend surgical intervention for main duct dilation >10 mm, cyst enlargement, and recurrent pancreatitis; this patient met the latter two criteria^[^3,4^]^.

Laparoscopic distal pancreatectomy (LDP) was used in line with the gold standard^[^11^]^. A systematic review by Venkat *et al* has shown that LDP provides significant perioperative advantages over open approaches without compromising oncologic outcomes^[^14^]^.

The reported case showed complete removal of microinvasive carcinoma, demonstrating the efficacy of laparoscopic resection. Although younger patients may experience a better prognosis post-resection, invasive features remain oncologically significant^[^13,15^]^.

Pancreatic intraepithelial neoplasia (PanIN) is classified by cytologic and architectural criteria; PanIN-3 (high-grade) represents the immediate precursor to invasive pancreatic ductal adenocarcinoma^[^16^]^. The presence of multifocal PanIN-3 in our patient highlights advanced neoplastic progression. Retrospective data from Ohio State University showed that resected high-grade PanIN offers a survival advantage, with median survival of approximately 37 months compared with 11 months in cases without PanIN^[^17^]^. Early detection of PDAC measuring ≤1 cm, often evolving from PanIN-3, is linked to better survival rates^[^18^]^. This highlights the importance of early lesion detection, especially for young patients with multifocal PanIN-3, as well as the need for long-term surveillance post-resection to monitor recurrence and improve outcomes.

The combination of young age, microinvasive disease, and multifocal PanIN-3 justified genetic evaluation. While young age alone does not meet standard referral criteria, the complex pathology warrants assessment for hereditary syndromes, including Peutz–Jeghers syndrome, Lynch syndrome, and BRCA-related syndromes^[^9,19^]^.

## Conclusion

This case underscores the necessity for high clinical suspicion of IPMN in young patients presenting with idiopathic recurrent pancreatitis. International guidelines lack differentiation of approaches according to patient’s age, and published studies focus only on elderly patients. Although IPMN in patients under 40 is uncommon^[^13^]^, specific cohorts exhibit aggressive features and can benefit from appropriate intervention. The selection of minimally invasive surgery aligns with best practices, combining safety and oncologic efficacy^[^14,20^]^. Given the potential for early malignant transformation, surgical resection in young patients should not be delayed^[^2,21^]^.

Taking these factors into account, the importance of genetic evaluation becomes essential^[^9^]^. It can affect surveillance plans, extend screening, and provide valuable information for specific risk models.

In conclusion, the convergence of young age, radiological evolution, recurrent pancreatitis, and a microinvasive lesion makes this a case of considerable interest. We advocate for an expedited investigation and a lower threshold for surgical consultation in young patients with IPMN exhibiting any worrisome features, even in the absence of classic high-risk stigmata. This case contributes to the evolving understanding of IPMN in younger populations and supports ongoing refinement of diagnostic and management guidelines to better address this understudied population.

## Data Availability

Not applicable.
